# The COVID‐19 lockdown and psychological distress among Italian parents: Influence of parental role, parent personality, and child difficulties

**DOI:** 10.1002/ijop.12755

**Published:** 2021-03-15

**Authors:** Cristina Mazza, Daniela Marchetti, Eleonora Ricci, Lilybeth Fontanesi, Serena Di Giandomenico, Maria Cristina Verrocchio, Paolo Roma

**Affiliations:** ^1^ Department of Neuroscience, Imaging and Clinical Sciences G. d'Annunzio University of Chieti‐Pescara Chieti Italy; ^2^ Department of Psychological Health and Territorial Sciences, G. d'Annunzio University of Chieti‐Pescara Chieti Italy; ^3^ Department of Humanities University of Urbino Carlo Bo Urbino Italy; ^4^ Department of Human Neuroscience Sapienza University of Rome Rome Italy

**Keywords:** Personality traits, Child difficulties, GHQ‐12, SDQ‐P, BFI‐10

## Abstract

The Italian lockdown following the spread of COVID‐19 exposed residents to a long and unexpected period of managing offspring at home. Throughout this time, most parents continued to work remotely. The present research aimed at assessing multiple sociodemographic and psychological variables for parental well‐being during the lockdown. An online survey was administered from 6 to 11 April 2020. Respondents were 917 parents aged 23–67 years with up to six children, aged 3–13 years. The measures employed were: 14 demographic questions, the Big Five Inventory (BFI‐10), the Emotional Symptoms and Hyperactivity‐Inattention subscales of the Strength and Difficulties Questionnaire (SDQ‐P), and the General Health Questionnaire (GHQ‐12). Multiple moderated linear regression analyses were performed. Motherhood, higher levels of education, higher neuroticism, lower extroversion, and more child emotional and hyperactivity‐inattention symptoms were found to be significant predictors of parent distress. Furthermore, a significant two‐way interaction between child emotional problems and parent extroversion was found. Overall, parents showed high rates of psychological distress, signalling severe difficulties during the lockdown. Families with a child suffering from emotional and behavioural difficulties should immediately be detected by social services to activate support interventions to prevent chronic and amplified manifestations of these problems.

The COVID‐19 outbreak and the government‐mandated measures to manage it have exposed individuals to new living situations, with significant consequences for important life domains (e.g., work, interpersonal relationships, psychological well‐being). Among these measures, the lockdown has been particularly burdensome, due to its pervasiveness and lengthy duration. Studies on forced cohabitation during lockdowns have demonstrated that long periods of lockdown can have a huge impact on mental health, reducing well‐being (Brooks et al., 2020). In fact, studies on past pandemics have reported that long durations of quarantine are significantly associated with greater psychological distress. A recent study (Mazza, Ricci, Biondi, et al., 2020) on psychological distress during the COVID‐19 lockdown in Italy showed a high prevalence of psychological symptomatology in the general population. The results highlighted that the female gender and the personality domains of negative affect, and detachment were associated with higher levels of depression, anxiety, and stress. Furthermore, having an acquaintance infected with COVID‐19 was associated with increased depression and stress, whereas prior experience of stressful situations and a history of specific medical problems were associated with higher levels of depression and anxiety. Finally, those with a family member infected with COVID‐19 and young persons who had to work outside the home presented higher levels of anxiety and stress, respectively.

Multiple recent studies have demonstrated that the COVID‐19 lockdown has had a significant psychological impact on specific populations (e.g., doctors, nurses, psychiatric patients, overweight/obese individuals). With respect to parents, research has highlighted that one quarter of isolated parents—and an even greater percentage of parents living with children—show a high level of psychological distress during quarantine (see, e.g., Sprang & Silman, 2013). Despite this evidence, few studies (Bıkmazer et al., 2020; Mazza, Ricci, Marchetti, et al., 2020; Morelli et al., 2020; Spinelli et al., 2020; Yue et al., 2020) have investigated the mental health of parents during the COVID‐19 lockdown, specifically. Yue et al. (2020) demonstrated that home quarantine during the COVID‐19 emergency was associated with a variety of psychological symptoms in families. Specifically, the authors found high levels of psychological distress in both children and parents during the lockdown period, with 1.18% of parents reporting high levels of anxiety, 3.60% of parents experiencing moderate depression and 3.53% meeting the diagnostic criteria for post‐traumatic stress disorder (PTSD). Furthermore, recent research has highlighted that the health risks and preoccupations linked with COVID‐19 influence parents' distress levels and, consequently, children's well‐being (Spinelli et al., 2020).

Specific COVID‐19 and sociodemographic variables may be significant in predicting psychological distress in parents. According to Bıkmazer et al. (2020), being a mother and a younger parent, having an acquaintance diagnosed with COVID‐19, having a positive psychiatric history, and living with a child with moderate or high emotional distress are all associated with significant parent distress. Along these same lines, Yue et al. (2020) found that mothers showed higher levels of anxiety, depression, and stress compared to fathers, and that they were more prone to developing PTSD symptoms. Moreover, research conducted on the general population (Mazza, Ricci, Biondi, et al., 2020) has underlined that women are more psychologically vulnerable to the COVID‐19 lockdown—a finding that has also received support from longitudinal studies.

Another significant relation highlighted in the international literature connects age and psychological distress, showing that young age is associated with increased stress, specifically in relation to career and education. Thus, in the context of the COVID‐19 lockdown, it is not surprising that younger parents have reported more difficulties compared to older parents. It is likely that younger parents may particularly struggle with caring for offspring during the unexpected COVID‐19 situation, and this may contribute to their psychological distress (Menon et al., 2020). Moreover, it has been shown that parents with younger children are more psychologically vulnerable. Marchetti et al. (2020) found that having a large number of children, having younger children, and having children with special needs were the best predictors for parenting‐related exhaustion during the COVID‐19 lockdown.

Previous studies have highlighted that lower levels of monthly household income may represent a risk factor for developing psychological distress, and particularly PTSD symptoms (Yue et al., 2020). Furthermore, a recent meta‐analysis highlighted that the female gender and lower socioeconomic status are associated with an eventual onset of PTSD (Tang et al., 2017). Qiu et al. (2020) also highlighted that, during the peak of the COVID‐19 pandemic in China, higher educational levels were associated with greater psychological distress, as individuals with higher education tended to have more self‐awareness of their health. In particular, those who had to leave their home and take public transportation to work experienced the highest levels of distress.

Morelli et al. (2020) found that parents who presented higher levels of psychological distress saw their working situation deteriorate during the COVID‐19 lockdown; these were mostly single or divorced parents who had to singlehandedly manage their children at home during the lockdown, while continuing to work. Furthermore, Horesh et al. (2020) demonstrated that, compared to those who were married or in a relationship, single individuals expressed greater concern that people close to them would get infected; they also presented lower levels of psychological and social well‐being. The authors highlighted that participants with pre‐existing chronic conditions (e.g., diabetes, high blood pressure, asthma, arthritis) reported increased worry about infection and lower physical well‐being, compared to those with no such conditions. Indeed, Mazza, Ricci, Biondi, et al. (2020) found that participants with a history of stressful situations and medical problems reported higher levels of depression and anxiety.

Personality traits (i.e., conscientiousness, agreeableness, openness to experience, emotional stability, extraversion) may comprise a further significant factor in predicting parent distress. For example, research (Smith, 2010) has highlighted that parents with high levels of neuroticism (i.e., low levels of emotional stability) tend to show high levels of psychological distress and anxiety. More recently, Mazza, Ricci, Marchetti, et al. (2020) found that neuroticism represented an important risk factor for parents' mental health during the COVID‐19 pandemic. Specifically, high levels of parent neuroticism—characterised by patterns of worry, nervousness, emotional instability, and feelings of inadequacy—were found to increase parent psychological distress, mainly in terms of anxiety, depression, and social dysfunction. On the other hand, parents with high levels of extraversion (i.e., positive affectivity, energy, sociality) tend to be more talkative, active, social and optimistic, and to enjoy higher well‐being and lower levels of parent distress. These parents are typically warm, sensitive and responsive towards their children and engage in high levels of parent–child interaction for the purpose of schooling, play, and emotional support (Ortiz & Barnes, 2018). Indeed, a consistent body of research (for a meta‐analysis, see Prinzie et al., 2009) has suggested that personality features can impact parenting quality by promoting or obstructing responsive parenting. For example, parents who are extraverted, open to experience, agreeable, conscientious, and emotionally stable (i.e., low in neuroticism) are more likely to provide their children with warmth, clear expectations and consistent limit setting; conversely, parents with high levels of neuroticism tend to experience negative life events more severely and to be more focused on themselves and their own distress, and less responsive to their children's needs (Smith, 2010).

Parental neuroticism has also been shown to be related to increased child mental health problems. Amrock and Weitzman (2014) found associations between parental neuroticism and abnormal emotional symptoms in younger children, conduct disorder in older children, and hyperactivity in children of all ages. Furthermore, studies have highlighted that parent distress can also be explained by the association between parental personality traits and children's emotional and behavioural issues (see, e.g., Plotkin et al., 2014). For example, parents, who reported high levels of neuroticism tend to also report more emotional and behavioural problems in children. Overall, parent distress has been associated with child problem behaviours, in general, as well as child externalising and/or internalising behaviours, specifically. Among the parents of children with problem behaviours, researchers have found high levels of stress, depression, and negative emotions, including anger, grief, guilt and a sense of child‐rearing inadequacy (O'Leimat et al., 2019).

The lockdown and school closure imposed by the Italian government on 9 March 2020, following the uncontrolled spread of COVID‐19, exposed Italian residents to a long and unexpected period of managing offspring at home. Throughout this period, most parents continued to work remotely. Building on the aforementioned findings, the present study examined various sociodemographic and psychological variables impacting parental well‐being during the initial COVID‐19 outbreak in Italy, to outline the most at‐risk family profiles and propose joint initiatives to support them. The findings provide important insights into the association between parent personality traits, child behavioural and emotional problems, and parent distress in a high‐stress environment (e.g., the COVID‐19 pandemic).

## MATERIAL AND METHODS

### Procedures

A Qualtrics online survey was administered from 6 to 11 April 2020, 1 month into the COVID‐19 lockdown in Italy. Participants accessed the survey via a designated link that was disseminated through the main means of communication and social networks, in order to reach a large number of parents (both mothers and fathers, indiscriminately). All participants voluntarily responded to the anonymous questionnaire and indicated their informed consent within. The procedures were clearly explained, and participants could interrupt or quit the survey at any point without providing a reason. The study was approved by the local ethics committee (Board of the Department of Human Neuroscience, Faculty of Medicine and Dentistry, Sapienza University of Rome).

### Participants

A total of 917 parents participated in the survey. The inclusion criteria were: (a) aged 18 years or older and (b) having at least one child, aged 3–13 years, living with them during the lockdown. The sample was comprised of both mothers (85.2%) and fathers (14.8%), aged 23–67 years (*M* = 40.67, *SD* = 6.5), each with up to six children (*M* = 1.81, *SD* = .756) aged 3–13 years (*M* = 7.59, *SD* = 3.2) (Table 1). Most participants (*n* = 356, 38.8%) held a high school diploma and were employed (*n* = 771, 84.1%). Furthermore, most reported that they were continuing to work remotely from home (*n* = 339, 37%) and following the government advice to stay at home. Table 1 presents descriptive statistics, including all characteristics considered.

**TABLE 1:** Descriptive statistics of the sample parents

Characteristic	Group	n (%)
Educational level	Primary school diploma	3 (0.3)
	Middle school diploma	57 (6.2)
	High school diploma	356 (38.8)
	Graduate	307 (33.5)
	Postgraduate	194 (21.2)
Occupation	Unemployed	146 (15.9)
	Employed	771 (84.1)
Socioeconomic status	Low (0–15,000)	118 (12.9)
	Medium‐low (16,000–33,000)	418 (45.6)
	Medium‐high (34,000–55,000)	294 (32.1)
	High (over 55,000)	87 (9.5)
Citizenship	Italian	899 (98.0)
	Foreign	18 (2.0)
Marital status	Unmarried/widower	37 (4.0)
	Married/cohabitant	783 (85.4)
	Separated/divorced	97 (10.6)
Region	Less affected region	727 (79.3)
	More affected region	190 (20.7)
Condition (home/work)	Must go to work	143 (15.6)
	Working from home	339 (37.0)
	Can stay home/work activity stopped/unemployed	435 (47.4)
Previously infected with COVID‐19	Yes	2 (0.2)
	No	915 (99.8)
Loved one(s) (e.g., family member, friend, relative) infected with COVID‐19	Yes	223 (24.3)
	No	694 (75.7)
Current or past medical issue(s)	Yes	175 (19.1)
	No	742 (80.9)
Psychological support or psychotherapy	Yes, previously/currently	243 (26.5)
	No	674 (73.5)

### Measures

Based on the aforementioned literature, the following measures were administered, in addition to questionnaires designed to collect sociodemographic information and data related to specific COVID‐19 variables.

#### The 10‐item big five inventory

(BFI‐10; Rammstedt, 2007) was used to measure personality characteristics—in particular the Big Five dimensions (i.e., *Openness to Experience, Agreeableness, Extroversion, Neuroticism/Emotional Stability, Conscientiousness*)—on a 5‐point Likert scale ranging from 1 (*totally disagree*) to 5 (*totally agree*). In the present study, the Spearman–Brown coefficient was used to assess the internal consistency of all subscales, as this reliability coefficient is recommended for the assessment of two‐item subscales. All correlations were significant (*p* < .001), with a maximum value of *r*_*s*_ = .45 for the Neuroticism/Emotional Stability subscale.

#### *The strengths and difficulties* *Questionnaire‐Parent*

SDQ‐P (Goodman, 1997) was used to evaluate child hyperactivity‐inattention and emotional symptoms. The SDQ is an instrument that screens for developmental psychopathology. Each subscale (i.e., *Conduct Problems, Hyperactivity‐Inattention, Emotional Symptoms, Peer Problems*) consists of five items, rated on a 3‐point Likert scale ranging from 0 (*not true*) to 2 (*certainly true*). The present study used the Hyperactivity‐Inattention and Emotional Symptoms subscales, exclusively. These subscales showed good internal consistency in the study sample, with Cronbach's alphas of .76 and .70, respectively.

#### The general health Questionnaire‐12

GHQ‐12 (Goldberg & Williams, 1988) is a 12‐item self‐report questionnaire that is used to assess of non‐psychotic psychiatric conditions. Responses are measured on a 4‐point Likert scale ranging from 1 (*less than usual*) to 4 (*much more than usual*). In the present study, Cronbach's alpha was .84, indicating good reliability.

### Statistical analysis

A hierarchical moderated linear regression analysis was used to determine the best predictors for parental well‐being. Using the enter method, sociodemographic variables (i.e., age, education, employment, economic status, parental role, marital status, number of children), medical/psychological factors (i.e., health‐related problems, psychological treatment) and personality traits (i.e., extraversion, agreeableness, conscientiousness, emotional stability/neuroticism, openness to experience) were entered in step 1. COVID‐19 variables (i.e., region of residence, going to work, working at home, staying at home without working, and child emotional and hyperactivity‐inattention symptoms during the lockdown) were entered in step 2. Two‐way interactions between parents' personality traits and child emotional and hyperactivity‐inattention difficulties were enclosed in step 3. Blocks 2 and 3 were run using the stepwise method. Statistical analyses were conducted using the software package SPSS, version 25 (IBM Inc., Armonk, New York).

### Ethics statement

All procedures performed in the present study involving human participants were in accordance with the ethical standards of the institutional research committee and the 1964 Helsinki Declaration and its later amendments or comparable ethical standards. Informed consent was obtained from all individual adult participants.

## RESULTS

**Figure 1:**
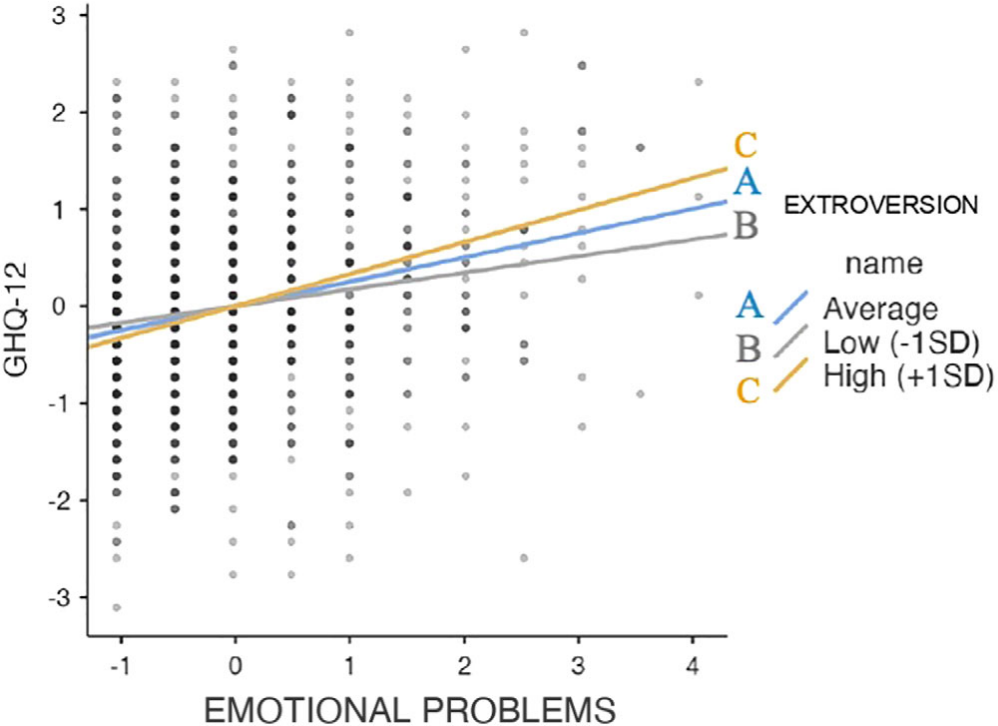
Simple slope analysis. [Colour figure can be viewed at wileyonlinelibrary.com].

The average GHQ‐12 total score (*M* = 19.34, *SD* = 5.90) in the present sample was higher than the mean reported in the Italian validation study (*M* = 10.43, *SD* = 5.42; Giorgi et al., 2014). The final moderated linear regression model was significant [*F*(18) = 11.628, *p* = <.001)] and accounted for approximately 20% of the variance in parental well‐being (*R* ^2^ = .189 [*Adj R* ^2^ = .173], *F‐*change = 6.371, *p* = .012). Motherhood, higher educational level, lower parent emotional stability (or higher neuroticism), lower parent extroversion, and more child emotional and hyperactivity‐inattention symptoms were found to be significant predictors of parent distress. Finally, the regression analysis revealed a significant two‐way interaction between child emotional problems and parent extroversion (Table 2). Simple slopes analyses specified that child emotional symptoms affected psychological distress in parents both when parents showed a higher (+1 *SD*) level of extroversion (*B* = .330, *p* = <.001) and when they showed a lower (−1 *SD*) level of extroversion (*B* = .172, *p* = <.001) (Figure 1).

**TABLE 2:** Hierarchical moderated linear regression model predicting parent distress

Predictor	b	SE	*β*	t	p
(Constant)	−.554	.243		−2.281	.023
Age	.005	.005	.036	1.062	.289
Educational level	**.082**	**.040**	**.073**	**2.067**	.039*
Unemployed_[ref. Employed]_	−.016	.088	−.006	−.185	.853
Unmarried/widower_[ref. Married]_	.037	.158	.007	.322	.816
Separated/divorced_[ref. Married]_	−.165	.104	−.051	−1.589	.112
Parental role_[ref. Motherhood]_	**−.192**	**.093**	**−.068**	**−2.067**	.039*
Number of children	.059	.041	.045	1.447	.148
Socioeconomic status	−.016	.043	−.014	−.383	.702
Current or past medical issue(s)_[ref. No]_	.122	.078	.048	1.567	.117
Previous or current psychological treatment_[ref. No]_	−.044	.072	−.020	−.618	.537
Conscientiousness (CON)	−.038	.031	−.038	−1.202	.230
Agreeableness (AGR)	.047	.032	.047	1.470	.142
Emotional stability (Neuroticism) (EMO)	**−.273**	**.033**	**−.273**	**−8.383**	<.001***
Extroversion (EXTR)	**−.062**	**.031**	**−.062**	**−2.013**	.044*
Openness to experience (OPEN)	−.042	.031	−.042	−1.347	.178
Child emotional problems after COVID‐19 (EP)	**.180**	**.032**	**.180**	**5.573**	<.001***
Child hyperactivity after COVID‐19 (HP)	**.097**	**.032**	**.098**	**2.985**	.003**
EP* EXTR	**.076**	**.030**	**.077**	**2.524**	.012*

Bold values are all significant for *p < 0.05, **p < 0.01 and ***p < 0.001.

Region of residence during the lockdown (*B* = .039, *p* = .211), going to work_[ref. staying at home without working]_ (*B* = −.014, *p* = .641), working at home_[ref. staying at home_ _without working_ (*B* = .031, *p* = .351), and all other interactions between child emotional and hyperactivity‐inattention symptoms and parent personality traits were excluded from the analysis.^1^

## DISCUSSION

Overall, parents of children aged 3–13 years, assessed 5 weeks after the school closure, showed very high rates of psychological distress, signalling severe difficulties during the lockdown. This higher distress level could predict future emotional and behavioural dysfunction, in both children and in parents (O'Leimat et al., 2019). Taken together, the present study found that motherhood, parent personality traits (i.e., high neuroticism, low extraversion), a high educational level, and more child emotional and hyperactivity‐inattention problems represented significant sociodemographic and psychological predictors of parental well‐being. Furthermore, child emotional symptoms were found to affect psychological distress in parents with both high and low levels of extroversion.

After parents' current working status was controlled for, motherhood emerged as a significant factor contributing to greater stress during the lockdown. This could be explained by two simultaneous factors: (a) the association between female gender and increased psychological distress and internalising symptoms; and (b) the sociocultural relation of the maternal role to childcare. Furthermore, this result is consistent with the findings of previous studies, showing a significant association between motherhood and parent distress (Bıkmazer et al., 2020), as well as research showing higher levels of anxiety, depression, and stress in mothers, compared to fathers and mothers' greater susceptibility to developing PTSD symptoms (Yue et al., 2020).

The present study also showed that parents with a high educational level experienced greater psychological distress. This result is aligned with that of Qiu et al. (2020), who found that individuals with a higher level of education were at greater risk for experiencing negative mental health outcomes, “probably because of high self‐awareness of their health” (p. 2).

The study also found that parent psychological distress was also explained by personality traits (i.e., high neuroticism, low extraversion). Neuroticism and extraversion are two of the most significant traits for predicting subjective well‐being. In particular, the relationship between neuroticism (i.e., sadness, anxiety, irritability, emotional instability) and internalising symptoms (i.e., depression, anxiety) is well documented. For instance, highly neurotic individuals become moody, angry, worried, and frustrated easily, and they tend to experience negative life events more severely. Furthermore, they are more vulnerable to influence and may readily become emotionally exhausted and depersonalised. It should be noted that there is an overlap between measures of neuroticism and some mental health outcomes: most of the items that define neuroticism are also characteristic of depression and anxiety; for this reason, it can be difficult to correctly interpret associations with these disorders. However, several longitudinal studies (see, e.g., Spijker et al., 2007) have shown that, after shared items and subjects' previous depressive status are controlled for, significant associations remain between the dimension of neuroticism and measures of depression.

The present results showed that parents with low extraversion (i.e., warmth, gregariousness, assertiveness, energy) tended to report higher levels of parent distress. The trait of neuroticism may contribute to a possible explanation for the finding. Individuals who are highly neurotic and introverted (i.e., reserved, withdrawn, inhibited) might present less adaptive coping strategies in reaction to potentially stressful events, and thereby experience more psychological distress. Furthermore, the present result is in line with the findings of Macía et al. (2020), who showed that low levels of neuroticism and high levels of extraversion related to positive health outcomes, and seemed to comprise protective factors with respect to mental health in cancer patients. Finally, the result is aligned with that of Mulsow et al. (2002), who found that high levels of extraversion, agreeableness, and emotional stability (i.e., low levels of neuroticism) were associated with lower levels of parenting stress in mothers of newborns, thereby decreasing the likelihood that these mothers would experience chronic distress after 2 years.

The present study also provided empirical support for the assumption that having children with psychological problems is detrimental for parents' mental well‐being. High levels of stress and depression have been reported among families of children with disabilities, with parents reporting difficult emotions, including anger, grief, guilt, and a sense of child‐rearing inadequacy (O'Leimat et al., 2019). Clearly, child emotional and behavioural difficulties have a significant impact on parent distress. In fact, Podolski and Nigg (2001) found that parents of children with hyperactivity‐inattention and oppositional‐conduct problems showed more distress and dissatisfaction in their parental role compared to parents in a control group. Moreover, a recent study (Romero et al., 2020) on the impact of the COVID‐19 lockdown on children in Spain found that parenting distress was directly and positively related to children's emotional problems, as well as hyperactivity and conduct problems.

A further finding of the present study was that child emotional problems influenced the psychological distress of parents with both high and low levels of extraversion. Previous studies have shown that “the multifactorial nature of extraversion may explain the differential associations reported between this construct and various aspects of parenting behavior” (Clark et al., 2000, p. 280). It is worth noting that the dimension of extraversion includes aspects that are beneficial to parenting (i.e., warmth, sociability, etc.), as well as aspects that may result in dysfunctional parenting behaviour (i.e., an authoritarian style). Moreover, the present results are in line with those of a recent study on the association between parent personality, parent stress, and child socioemotional development (Ortiz & Barnes, 2018), showing that the children of mothers with low levels of extraversion and greater parent distress were likely to have more internalising problems. Specifically, it seems that parental personality might play an important role in determining the intensity of child emotional and behavioural problems, depending on the personality features of the individual parent (Plotkin et al., 2014), which might, in turn, influence the parents' parenting style and response to stressors.

Overall, in line with the literature, the psychological distress found in the present study seemed to result from multidimensional factors. Neuroticism was found to predict child behavioural problems, both independently and as a mediator linked to maternal depression. In turn, maternal neuroticism was found to significantly related to mothers' emotional responses to their children's emotional and behavioural difficulties. Scalzo et al. (2005) highlighted that maternal neuroticism, depression, and self‐reported well‐being significantly related to mothers' responses to their children's problems. Specifically, mothers who scored high on neuroticism tended to respond to their children's problems with more negative emotionality, compared to mothers who scored low on neuroticism. Indeed, when parents experience high levels of psychological distress, their ability to respond sensitively and effectively to their children's emotional needs is limited; this may restrict their ability to promote self‐regulation and lead to more intense and pervasive child behaviour problems. It is important to note that, during the COVID‐19 lockdown, parents were unable to rely on support from a relational network (e.g., family members and teachers), and those with a low level of extroversion may have experienced this as a greater challenge.

To prevent chronic and amplified manifestations of these problems, families with children suffering from emotional and behavioural difficulties should immediately be identified by mental health and social services, to activate family‐based support interventions. As suggested by Fontanesi et al. (2020), the governments of affected countries (including Italy) should: (a) promote systematic preventive and promotion activities aimed at organising and increasing resources to support families; (b) establish multidisciplinary teams to deliver a rapid assessment of families and groups at higher risk, both during and after any lockdown measure; and (c) train professionals on the effects of lockdown and isolation on parenting and child well‐being. Taken together, the results of the present study highlight the importance of assessing parental personality characteristics and their potential interaction with children's emotional problems.

A main limitation of the present research is that the sample was composed of mostly mothers, thereby reducing the generalizability of the results to fathers. Indeed, as the study was conducted online, it was not possible to determine whether both parents in a family unit—or only one parent—participated in completing the measures. A further limitation pertains to the great variability in family types, and the fact that neither the sexual orientation nor the ethnicity of parents was analysed. Finally, child emotional and behavioural functioning was rated by parents, and not directly through child observation. Thus, future research should implement a wide personality assessment and suggest preventive measures targeting families who may already receive local mental health assistance for behavioural and/or emotional difficulties. Importantly, support interventions should be directed at parents of children who are already known to present personality dysfunctions.

## Endnote

^1^
HP*CON (*B* = .016, *p* = .591); HP*AGR (*B* = .029, *p* = .345); HP*EMO (*B* = −.012, *p* = .706); HP*EXTR (*B* = .025, *p* = .425); HP*OPEN (*B* = −.041, *p* = .181); PE*CON (*B* = −.025, *p* = .414); PE*AGR (*B* = .054, *p* = .078); PE*EMO (*B* = −.020, *p* = .517); PE*OPEN (*B* = .008, *p* = .782).
